# 2,4-Dimethyl­anilinium chloride

**DOI:** 10.1107/S1600536810020568

**Published:** 2010-06-05

**Authors:** Ji-Yuan Yao

**Affiliations:** a Ordered Matter Science Research Center, College of Chemistry and Chemical Engineering, Southeast University, Nanjing 210096, People’s Republic of China

## Abstract

In the crystal structure of the title compound, C_8_H_12_N^+^·Cl^−^, all H atoms bonded to the ammonium N atom are hydrogen bonded to the chloride ions, with N⋯Cl distances in the range 3.080 (2)–3.136 (2) Å, resulting in 16-membered macrocyclic rings involving four formula units of the title compound.

## Related literature

For background to phase transition materials see: Li *et al.* (2008 ▶); Zhang *et al.* (2009 ▶).
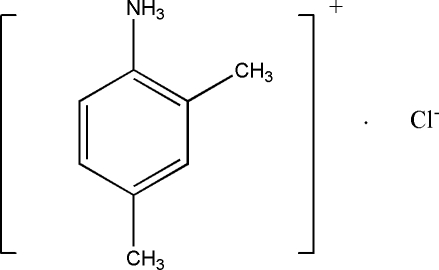


## Experimental

### 

#### Crystal data

C_8_H_12_N^+^·Cl^−^
*M* *_r_* = 157.64
Monoclinic, 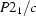

*a* = 9.4739 (19) Å
*b* = 9.894 (2) Å
*c* = 9.6709 (19) Å
β = 96.31 (3)°
*V* = 901.0 (3) Å^3^
*Z* = 4
Mo *K*α radiation
μ = 0.35 mm^−1^
*T* = 293 K
0.4 × 0.3 × 0.2 mm

#### Data collection

Rigaku Mercury2 diffractometer
Absorption correction: multi-scan (*CrystalClear*; Rigaku, 2005 ▶) *T*_min_ = 0.880, *T*_max_ = 0.932
9081 measured reflections
2068 independent reflections
1585 reflections with *I* > 2σ(*I*)
*R*_int_ = 0.038

#### Refinement

*R*[*F* ^2^ > 2σ(*F* ^2^)] = 0.047
*wR*(*F* ^2^) = 0.150
*S* = 1.01
2068 reflections
91 parameters
H-atom parameters constrained
Δρ_max_ = 0.38 e Å^−3^
Δρ_min_ = −0.28 e Å^−3^

### 

Data collection: *CrystalClear* (Rigaku, 2005 ▶); cell refinement: *CrystalClear*; data reduction: *CrystalClear*; program(s) used to solve structure: *SHELXS97* (Sheldrick, 2008 ▶); program(s) used to refine structure: *SHELXL97* (Sheldrick, 2008 ▶); molecular graphics: *SHELXTL* (Sheldrick, 2008 ▶); software used to prepare material for publication: *PRPKAPPA* (Ferguson, 1999 ▶).

## Supplementary Material

Crystal structure: contains datablocks I, global. DOI: 10.1107/S1600536810020568/pv2285sup1.cif

Structure factors: contains datablocks I. DOI: 10.1107/S1600536810020568/pv2285Isup2.hkl

Additional supplementary materials:  crystallographic information; 3D view; checkCIF report

## Figures and Tables

**Table 1:** Hydrogen-bond geometry (Å, °)

*D*—H⋯*A*	*D*—H	H⋯*A*	*D*⋯*A*	*D*—H⋯*A*
N1—H1*A*⋯Cl1^i^	0.89	2.27	3.136 (2)	164
N1—H1*B*⋯Cl1^ii^	0.89	2.27	3.128 (2)	163
N1—H1*C*⋯Cl1	0.89	2.20	3.080 (2)	170

Symmetry codes: (i) 
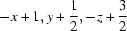
; (ii) 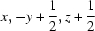.

**Fig. 1.**
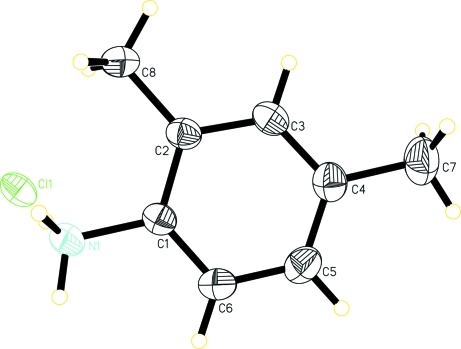
The molecular structure of the title compound, showing the atomic numbering scheme. Displacement ellipsoids are drawn at the 30% probability level.

**Fig. 2.**
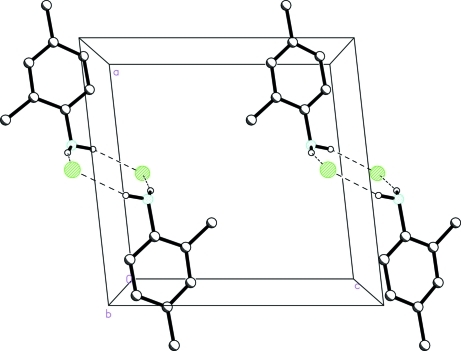
A view of the packing of the title compound, showing H-bonded bands along the c-axis; dashed lines indicate hydrogen bonds.

## supplementary crystallographic information

### Comment

There has been interest in the study of phase transition materials, including organic ligands, metal-organic coordination compounds, organic-inorganic hybrids, etc. (Li *et al.*, 2008; Zhang *et al.*, 2009). Exploring the phase transition materials, the dielectric properties of the title compound, have been investigated in my laboratory. Unfortunately, there was no distinct anomaly observed from 93 K to 380 K, (m.p. 408 K-410 K). In this article, the crystal structure of the title compound has been presented.

The asymmetric unit of the title compound contains a 2,4-dimethylanilinium cation and a chloride anion (Fig. 1). The non-H atoms of the 2,4-dimethylanilinium cation are essentially coplanar. In the crystal structure, all hydrogen atoms bonded to the ammonium nitrogen (N1) are hydrogen bonded to the chloride ions with N···Cl distances in the range 3.080 (2) - 3.136 (2) Å; four formula units of the title compound are hydrogen bonded to form sixteen membered macrocyclic rings in the *bc*-plane (Tab. 1, Fig. 2).

### Experimental

The title compound was prepared by the reaction of 2,4-dimethylbenzenamine (1.21 g, 10 mmol) and hydrochloric acid solution (1.01 g, 10 mmol) in 30 ml methanol. The reaction mixture was filtered and left at room temperature for 4 days. Colorless crystals were obtained by slow evaporation.

### Refinement

Positional parameters of all H atoms were calculated geometrically and were allowed to ride on the atoms to which they are bonded, with N—H = 0.89 Å and C—H = 0.93 and 0.96 Å, for aryl and methyl type H-atoms, respectively, *U*_iso_(H) = 1.2 to 1.5*U*_eq_(C/N).

### Crystal data

**Table:**

C_8_H_12_N^+^·Cl^−^	*F*(000) = 336
*M**_r_* = 157.64	*D*_x_ = 1.162 Mg m^−^^3^
Monoclinic, *P*2_1_/*c*	Mo *K*α radiation, λ = 0.71073 Å
Hall symbol: -P 2ybc	Cell parameters from 7851 reflections
*a* = 9.4739 (19) Å	θ = 3.2–27.5°
*b* = 9.894 (2) Å	µ = 0.35 mm^−^^1^
*c* = 9.6709 (19) Å	*T* = 293 K
β = 96.31 (3)°	Prism, colourless
*V* = 901.0 (3) Å^3^	0.4 × 0.3 × 0.2 mm
*Z* = 4	

### Data collection

**Table:**

Rigaku Mercury2 diffractometer	2068 independent reflections
Radiation source: fine-focus sealed tube	1585 reflections with *I* > 2σ(*I*)
graphite	*R*_int_ = 0.038
Detector resolution: 13.6612 pixels mm^-1^	θ_max_ = 27.5°, θ_min_ = 3.5°
CCD_Profile_fitting scans	*h* = −12→12
Absorption correction: multi-scan (*CrystalClear*; Rigaku, 2005)	*k* = −12→12
*T*_min_ = 0.880, *T*_max_ = 0.932	*l* = −12→12
9081 measured reflections	

### Refinement

**Table:**

Refinement on *F*^2^	Primary atom site location: structure-invariant direct methods
Least-squares matrix: full	Secondary atom site location: difference Fourier map
*R*[*F*^2^ > 2σ(*F*^2^)] = 0.047	Hydrogen site location: inferred from neighbouring sites
*wR*(*F*^2^) = 0.150	H-atom parameters constrained
*S* = 1.01	*w* = 1/[σ^2^(*F*_o_^2^) + (0.0863*P*)^2^ + 0.190*P*] where *P* = (*F*_o_^2^ + 2*F*_c_^2^)/3
2068 reflections	(Δ/σ)_max_ < 0.001
91 parameters	Δρ_max_ = 0.38 e Å^−^^3^
0 restraints	Δρ_min_ = −0.28 e Å^−^^3^

### Special details

**Table:**

Geometry. All e.s.d.'s (except the e.s.d. in the dihedral angle between two l.s. planes) are estimated using the full covariance matrix. The cell e.s.d.'s are taken into account individually in the estimation of e.s.d.'s in distances, angles and torsion angles; correlations between e.s.d.'s in cell parameters are only used when they are defined by crystal symmetry. An approximate (isotropic) treatment of cell e.s.d.'s is used for estimating e.s.d.'s involving l.s. planes.
Refinement. Refinement of *F*^2^ against ALL reflections. The weighted *R*-factor *wR* and goodness of fit *S* are based on *F*^2^, conventional *R*-factors *R* are based on *F*, with *F* set to zero for negative *F*^2^. The threshold expression of *F*^2^ > σ(*F*^2^) is used only for calculating *R*-factors(gt) *etc*. and is not relevant to the choice of reflections for refinement. *R*-factors based on *F*^2^ are statistically about twice as large as those based on *F*, and *R*- factors based on ALL data will be even larger.

### Fractional atomic coordinates and isotropic or equivalent isotropic displacement parameters (Å^2^)

**Table:**

	*x*	*y*	*z*	*U*_iso_*/*U*_eq_	
N1	0.61300 (18)	0.39383 (17)	0.84555 (17)	0.0480 (4)	
H1A	0.5798	0.4779	0.8370	0.072*	
H1B	0.5968	0.3608	0.9279	0.072*	
H1C	0.5694	0.3426	0.7783	0.072*	
C1	0.7662 (2)	0.39422 (18)	0.8348 (2)	0.0436 (5)	
C3	0.9610 (2)	0.4527 (2)	0.7141 (2)	0.0538 (5)	
H3A	0.9972	0.4933	0.6388	0.065*	
C2	0.8145 (2)	0.4537 (2)	0.7185 (2)	0.0473 (5)	
C6	0.8563 (2)	0.3347 (2)	0.9380 (2)	0.0533 (5)	
H6A	0.8205	0.2946	1.0138	0.064*	
C4	1.0551 (2)	0.3944 (2)	0.8157 (2)	0.0562 (5)	
C5	1.0013 (3)	0.3352 (2)	0.9280 (3)	0.0609 (6)	
H5A	1.0628	0.2951	0.9977	0.073*	
C8	0.7157 (3)	0.5163 (3)	0.6043 (2)	0.0658 (7)	
H8A	0.6591	0.5843	0.6427	0.099*	
H8B	0.6547	0.4478	0.5600	0.099*	
H8C	0.7700	0.5567	0.5370	0.099*	
C7	1.2133 (3)	0.3985 (3)	0.8049 (3)	0.0834 (9)	
H7A	1.2629	0.3545	0.8843	0.125*	
H7B	1.2440	0.4908	0.8018	0.125*	
H7C	1.2332	0.3529	0.7216	0.125*	
Cl1	0.49251 (6)	0.19132 (5)	0.62110 (6)	0.0603 (2)	

### Atomic displacement parameters (Å^2^)

**Table:**

	*U*^11^	*U*^22^	*U*^33^	*U*^12^	*U*^13^	*U*^23^
N1	0.0525 (10)	0.0481 (9)	0.0456 (9)	−0.0007 (7)	0.0146 (7)	−0.0017 (7)
C1	0.0499 (11)	0.0389 (10)	0.0434 (10)	0.0000 (8)	0.0116 (8)	−0.0035 (7)
C3	0.0564 (12)	0.0585 (13)	0.0494 (11)	−0.0015 (10)	0.0185 (9)	−0.0012 (9)
C2	0.0545 (12)	0.0456 (10)	0.0436 (10)	0.0024 (8)	0.0126 (8)	0.0004 (8)
C6	0.0638 (14)	0.0515 (12)	0.0454 (11)	0.0031 (9)	0.0090 (10)	0.0047 (9)
C4	0.0512 (12)	0.0599 (13)	0.0583 (12)	0.0030 (10)	0.0098 (10)	−0.0126 (10)
C5	0.0619 (14)	0.0652 (14)	0.0542 (12)	0.0134 (11)	−0.0002 (10)	−0.0005 (11)
C8	0.0661 (14)	0.0782 (16)	0.0543 (13)	0.0066 (12)	0.0122 (11)	0.0231 (12)
C7	0.0516 (14)	0.111 (2)	0.0883 (19)	0.0052 (14)	0.0084 (13)	−0.0130 (17)
Cl1	0.0705 (4)	0.0570 (4)	0.0571 (4)	−0.0126 (2)	0.0235 (3)	−0.0140 (2)

### Geometric parameters (Å, °)

**Table:**

N1—C1	1.466 (3)	C6—H6A	0.9300
N1—H1A	0.8900	C4—C5	1.380 (3)
N1—H1B	0.8900	C4—C7	1.514 (3)
N1—H1C	0.8900	C5—H5A	0.9300
C1—C6	1.372 (3)	C8—H8A	0.9600
C1—C2	1.391 (3)	C8—H8B	0.9600
C3—C4	1.379 (3)	C8—H8C	0.9600
C3—C2	1.394 (3)	C7—H7A	0.9600
C3—H3A	0.9300	C7—H7B	0.9600
C2—C8	1.500 (3)	C7—H7C	0.9600
C6—C5	1.388 (3)		
			
C1—N1—H1A	109.5	C3—C4—C5	118.2 (2)
C1—N1—H1B	109.5	C3—C4—C7	120.4 (2)
H1A—N1—H1B	109.5	C5—C4—C7	121.4 (2)
C1—N1—H1C	109.5	C4—C5—C6	120.7 (2)
H1A—N1—H1C	109.5	C4—C5—H5A	119.7
H1B—N1—H1C	109.5	C6—C5—H5A	119.7
C6—C1—C2	122.39 (19)	C2—C8—H8A	109.5
C6—C1—N1	119.27 (17)	C2—C8—H8B	109.5
C2—C1—N1	118.33 (18)	H8A—C8—H8B	109.5
C4—C3—C2	123.4 (2)	C2—C8—H8C	109.5
C4—C3—H3A	118.3	H8A—C8—H8C	109.5
C2—C3—H3A	118.3	H8B—C8—H8C	109.5
C1—C2—C3	116.01 (19)	C4—C7—H7A	109.5
C1—C2—C8	122.39 (19)	C4—C7—H7B	109.5
C3—C2—C8	121.60 (19)	H7A—C7—H7B	109.5
C1—C6—C5	119.3 (2)	C4—C7—H7C	109.5
C1—C6—H6A	120.3	H7A—C7—H7C	109.5
C5—C6—H6A	120.3	H7B—C7—H7C	109.5

### Hydrogen-bond geometry (Å, °)

**Table:**

*D*—H···*A*	*D*—H	H···*A*	*D*···*A*	*D*—H···*A*
N1—H1A···Cl1^i^	0.89	2.27	3.136 (2)	164
N1—H1B···Cl1^ii^	0.89	2.27	3.128 (2)	163
N1—H1C···Cl1	0.89	2.20	3.080 (2)	170

Symmetry codes: (i) −*x*+1, *y*+1/2, −*z*+3/2; (ii) *x*, −*y*+1/2, *z*+1/2.

## References

[bb1] Ferguson, G. (1999). *PRPKAPPA* University of Guelph, Canada.

[bb2] Li, X. Z., Qu, Z. R. & Xiong, R. G. (2008). *Chin. J. Chem.* **11**, 1959–1962.

[bb3] Rigaku (2005). *CrystalClear* Rigaku Corporation, Tokyo, Japan.

[bb4] Sheldrick, G. M. (2008). *Acta Cryst.* A**64**, 112–122. 10.1107/S0108767307043930 18156677

[bb5] Zhang, W., Chen, L. Z., Xiong, R. G., Nakamura, T. & Huang, S. D. (2009). *J. Am. Chem. Soc.* **131**, 12544–12545. 10.1021/ja905399x 19685869

